# Isolation and Annotation of the Genome Sequences of Bacteriophages InvictusManeo (Subcluster K5) and Netyap (Subcluster L2)

**DOI:** 10.1128/mra.00160-22

**Published:** 2022-05-10

**Authors:** Maximiliano F. Flota, Véronique A. Delesalle, Caitlyn R. Moss, Davis S. Beeson, Andrew M. Bogatkevich, Clarissa C. Burgess, Noemi Carretero Lazcano, Jayda A. Carroll-Deaton, Hayden A. Deprill, Ivy S. Dickens, Maria D. Gainey, Sophia G. Gierszal, Avery A. Goff, Brooke K. Harris, John B. LeBrun, Jim Lin, Molly R. McLaughlin, Brian C. Metts, Kristen L. O’Rear, Maria A. Osorio Hernandez, Isabella E. Raieta, Erica D. Schmidt, Thomas D. Sinkway, Kimberly S. Sok, Michael A. Ulrich, Isaiah T. Velez, Jamie R. Wallen, Ayden R. Wardius, Christine A. Byrum

**Affiliations:** a Department of Biology, College of Charleston, Charleston, South Carolina, USA; b Department of Biological Sciences, Gettysburg College, Gettysburg, Pennsylvania, USA; c Department of Chemistry and Physics, Western Carolina University, Cullowhee, North Carolina, USA; Loyola University Chicago

## Abstract

The mycobacteriophages InvictusManeo (K5 subcluster) and Netyap (L2 subcluster) were isolated from soils in Cullowhee Creek, Cullowhee, North Carolina. Both exhibit *Siphoviridae* morphology and infect Mycobacterium smegmatis mc^2^155. The InvictusManeo genome is 61,147 bp and contains 96 predicted protein-coding genes, whereas the Netyap genome is 76,366 bp with 131 predicted protein-coding genes.

## ANNOUNCEMENT

**FIG 1:**
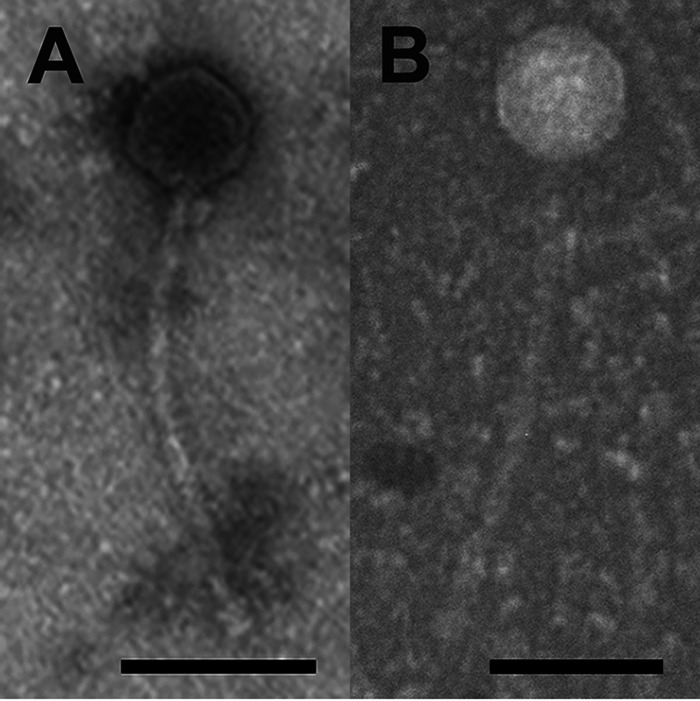
InvictusManeo (A) and Netyap (B) morphology examined using transmission electron microscopy. High-titer lysates placed on Formvar-coated copper grids were negatively stained with 1% uranyl acetate (2). Both phages exhibit *Siphoviridae* morphology. Scale bars, 100 nm.

The mycobacteriophages InvictusManeo and Netyap were isolated from single soil samples collected near Cullowhee Creek in Cullowhee, North Carolina (Table 1). Their genomes were analyzed in the Science Education Alliance-Phage Hunters Advancing Genomics and Evolutionary Science (SEA-PHAGES) Program of the Howard Hughes Medical Institute (HHMI) (1) to increase understanding of viral evolution and diversity. Both viruses infect Mycobacterium smegmatis mc^2^155 and were isolated using enrichment at 37°C followed by two purification/amplification cycles in 7H9 top agar (SEA-PHAGES Phage Discovery Guide protocol) (2). Electron microscopy revealed that they exhibit *Siphoviridae* morphotypes (Fig. 1).

**TABLE 1:** Characteristics of the InvictusManeo and Netyap bacteriophages

Parameter	Data for phage:	
	InvictusManeo	Netyap
GenBank accession no.	MZ958747	MW578835
SRA accession no.	SRX11158994	SRX11158997
Genome size (bp)	61,147	76,366
Collection location coordinates	35.316432N, 83.165618W	35.310051N, 83.187270W
GC content (%)	65.6	58.9
Coverage (×)	250	219
No. of predicted protein-coding genes	96	131
No. of tRNAs	1	12
No. of tmRNAs	0	0
Plaque size (mm) (*n* = 10)		
Range	1.9–4.7	1.3–2.5
Mean	3.0	1.9
Capsid size (nm) (*n* = 5 [InvictusManeo] or 6 [Netyap])		
Range	65–70	76–81
Mean	67.0	78.5
Tail length (nm) (*n* = 5 [InvictusManeo] or 6 [Netyap])		
Range	112–119	270–318
Mean	115.8	287.3

For sequencing, DNA was extracted from high-titer lysates with the Promega Wizard DNA cleanup system, followed by library preparation with a NEBNext Ultra II DNA library prep kit. The Western Carolina University Biotechnology Core performed shotgun sequencing on an Illumina MiSeq system (Nano v2 reagents) (3), producing 224,694 (InvictusManeo) and 238,646 (Netyap) single-end 150-bp reads. Reads were assembled into single contigs with Newbler v2.9 (4) and verified using Consed v29.0 (5) as described by Russell (3). Both genomes are linear with 3′ sticky overhangs (InvictusManeo, 5′-CTCAGTGGCAT-3′; Netyap, 5′-TCGATCAGCC-3′) and were annotated using the PECAAN workflow tool (6) and then transferred to DNA Master v5.22.2 (https://phagesdb.org/DNAMaster). GeneMark v2.5 (7), GLIMMER v3.02 (8), and Starterator v1.1 (9) were utilized to refine start sites, and comparative analysis was performed using Phamerator (10). Functional assignments were made with BLASTp v2.9 (11), HHpred (12), TMHMM v2.0 (https://services.healthtech.dtu.dk/service.php?TMHMM-2.0), TOPCONS v2 (13), and the NCBI Conserved Domain Database (CDD) (14), while tRNAs and transfer-messenger RNAs (tmRNAs) were identified using ARAGORN v1.2.38 (15) and tRNAscan-SE v3.0 (16). All programs used default parameters.

Mycobacteriophages sharing >50% nucleotide sequence similarity are categorized as members of the same cluster and are divided into subclusters based on average nucleotide identity (17, 18). InvictusManeo is a K5 subcluster member, with a genome containing 96 predicted protein-coding genes (36 with assigned putative functions), 3 orphan genes (*gp93* to *gp95*), and 1 tRNA gene. Genes include those for typical structural and assembly proteins, a lysis cassette (lysin A, lysin B, and holin), and the lysogeny-regulating proteins serine integrase (restricted to seven K5 subcluster members) and immunity repressor. Although their functional roles are unknown, gene products 1, 44, 50, 51, 54, and 92 may warrant further investigation; conserved in all K5 subcluster members, they are absent in other bacteriophages, which suggests that they may play crucial roles in the K5 subcluster. Whole-genome BLASTn alignment (11) of InvictusManeo to other bacteriophages indicates high levels of similarity to the K5 bacteriophages Collard (GenBank accession number NC_051593) (97.63% identity with 100% coverage), Kratio (GenBank accession number NC_028947) (99.02% identity with 97% coverage), and Larva (GenBank accession number NC_023724) (97.78% identity with 95% coverage).

The Netyap genome (L2 subcluster) contains 131 predicted protein-coding genes (55 with assigned putative functions), 2 orphan genes (*gp53* and *gp136*), and 12 tRNA genes. Lysin A and lysin B are present, as well as the lysogeny-regulating proteins tyrosine integrase, immunity repressor, Cro, and excise, but holin was not detected. Gene products 21, 24, 25, 34, 48, and 51 (no known functions) may interest L2 phage investigators, because these occur only in the L2 subcluster and are conserved in all members. Although it is a temperate phage (lysogens can be readily isolated) (19), it is noteworthy that Netyap and most other L2 subcluster members form clear, lytic plaques. Finally, whole-genome BLASTn alignments (11) reveal a high level of nucleotide sequence conservation between Netyap and Faith1 (GenBank accession number NC_015584) (99.97% identity with 99% coverage).

### Data availability.

Individual GenBank and SRA numbers are listed in Table 1.
